# The Effect of Apolipoprotein E ε4 (APOE ε4) on Visuospatial Working Memory in Healthy Elderly and Amnestic Mild Cognitive Impairment Patients: An Event-Related Potentials Study

**DOI:** 10.3389/fnagi.2017.00145

**Published:** 2017-05-17

**Authors:** Li-Hua Gu, Jiu Chen, Li-Juan Gao, Hao Shu, Zan Wang, Duan Liu, Yan-Na Yan, Shi-Jiang Li, Zhi-Jun Zhang

**Affiliations:** ^1^Department of Neurology, Affiliated ZhongDa Hospital, School of Medicine, Southeast UniversityNanjing, China; ^2^Department of Psychology, Xinxiang Medical UniversityXinxiang, China; ^3^Department of Biophysics, Medical College of Wisconsin, MilwaukeeWI, United States

**Keywords:** apolipoprotein E, event-related potential, amnestic mild cognitive impairment, visuospatial working memory, P300

## Abstract

**Background:** Apolipoprotein E (APOE) ε4 is the only established risk gene for late-onset, sporadic Alzheimer’s disease (AD). Previous studies have provided inconsistent evidence for the effect of APOE ε4 status on the visuospatial working memory (VSWM).

**Objective:** The aim was to investigate the effect of APOE ε4 on VSWM with an event-related potential (ERP) study in healthy controls (HC) and amnestic mild cognitive impairment (aMCI) patients.

**Methods:** The study recorded 39 aMCI patients (27 APOE ε4 non-carriers and 12 APOE ε4 carriers) and their 43 matched controls (25 APOE ε4 non-carriers and 18 APOE ε4 carriers) with an 64-channel electroencephalogram. Participants performed an N-back task, a VSWM paradigm that manipulated the number of items to be stored in memory.

**Results:** The present study detected reduced accuracy and delayed mean correct response time (RT) in aMCI patients compared to HC. P300, a positive component that peaks between 300 and 500 ms, was elicited by the VSWM task. In addition, aMCI patients showed decreased P300 amplitude at the central–parietal (CP1, CPz, and CP2) and parietal (P1, Pz, and P2) electrodes in 0- and 1-back task compared to HC. In both HC and aMCI patients, APOE ε4 carriers showed reduced P300 amplitude with respect to non-carriers, whereas no significant differences in accuracy or RT were detected between APOE ε4 carriers and non-carriers. Additionally, standardized low-resolution brain electromagnetic tomography analysis (s-LORETA) showed enhanced brain activation in the right parahippocampal gyrus (PHG) during P300 time range in APOE ε4 carriers with respect to non-carriers in aMCI patients.

**Conclusion:** It demonstrated that P300 amplitude could predict VSWM deficits in aMCI patients and contribute to early detection of VSWM deficits in APOE ε4 carriers.

## Introduction

Alzheimer’s disease (AD) is the most common cause of dementia, with progressive cognitive impairment as the primary symptom. Mild cognitive impairment (MCI) represents a transitional stage between normal aging and dementia (Petersen et al., 1999; Laczó et al., 2009). Amnestic mild cognitive impairment (aMCI) refers to the amnestic form of MCI. It has been demonstrated that MCI patients convert to probable AD at a rate of approximately 10–15% per year (Grundman et al., 2004), while normal controls progress to dementia at a rate of 1–2% per year (Bischkopf et al., 2002). Thus, early detection of MCI patients might be crucial for slowing down its progression to AD. Humans possess three major apolipoprotein E (APOE) isoforms encoded by ε2, ε3, and ε4 alleles. APOE ε4 is the only established risk gene for late-onset, sporadic AD (Seripa et al., 2009). Previous investigations demonstrated the critical role of APOE ε4 in the onset and progression of AD. APOE ε4 carriers show more faster cognitive decline (Bretsky et al., 2003), atrophic hippocampal volume in magnetic resonance imaging (MRI) markers (Petersen et al., 2000; Shi et al., 2009), increased cerebral amyloid deposition (Klunk et al., 2004) and cerebral hypometabolism compared to non-carriers (Reiman et al., 2005).

Working memory (WM) is a cognitive system for temporary holding, processing and manipulation of information. Regarding WM, previous studies confirmed that it is related to information processing speed and executive function (Brown et al., 2012). Episodic and semantic memory were deemed as earliest cognitive impairments in aMCI patients in previous investigations (Hodges et al., 2006; Leyhe and Muller, 2009). However, recent studies reported that WM impairment might also be a sensitive biomarker for the early detection of aMCI (Saunders and Summers, 2011; Zheng et al., 2012). Previous studies demonstrated that visuospatial working memory (VSWM) deficits start near the beginning of the MCI phase (Twamley et al., 2006; Alescio-Lautier et al., 2007; Webster et al., 2014). In APP mouse models of AD, spatial working memory (SWM) assessed by Morris water maze test was the earliest observable cognitive domain to demonstrate deficits, followed by recognition memory (Alescio-Lautier et al., 2007; Webster et al., 2013, 2014). In recent years, the applications of functional MRI (fMRI) studies have made prominent advances in the investigations of MCI. Several fMRI investigations identified the presence of VSWM related functional brain activation changes in aMCI patients (Kochan et al., 2010; Alichniewicz et al., 2012, Mitolo et al., 2013; Lou et al., 2015). However, regarding the effect of APOE ε4 status on the VSWM, previous fMRI studies provide inconsistent evidence. A study reported that APOE ε4 carriers in cognitively intact adults show lesser activation in medial temporal lobes (MTL) compared to non-carriers during VSWM (Borghesani et al., 2008), whereas Alichniewicz et al. (2012) indicated that APOE ε4 status has no impact on brain activation during VSWM in healthy controls (HC) or aMCI patients. Another neuropsychological assessment revealed no influence of APOE polymorphisms on WM in cognitively intact adults (Ward et al., 2014). Thus, the present study aimed to investigate the effect of APOE ε4 status on VSWM with event-related potential (ERP).

ERP has an excellent temporal resolution and reflects instantly the summated excitatory postsynaptic potential (EPSP) and inhibitory postsynaptic potential (IPSP), primarily of pyramidal cells in the neocortex (Nunez and Srinivasan, 2006). The high temporal resolution of ERP enabled the understanding of the neural correlates of cognitive processing and functioning. The present investigation used the N-back paradigm to evaluate the effect of APOE ε4 status on VSWM. The load factor N can be adjusted to make the task more or less difficult (e.g., 0-back and 1-back). In previous ERP studies, P300, a positive component that peaks between 300 and 500 ms, was elicited by WM tasks (Watter et al., 2001; Nakao et al., 2012). According to neuropsychological opinion, classical P300 (P3b) was produced by updating operation of WM (Papageorgiou et al., 2002; Polich, 2007). A study with an auditory target detection task indicated decreased P300 amplitude and prolonged P300 latency in APOE ε4 carriers compared to non-carriers in cognitively intact adults (Irimajiri et al., 2010). The current ERP study was designed as a novel investigation on the impact of APOE ε4 status on VSWM in HC and aMCI patients.

The aim of the present study was to investigate the influence of APOE ε4 status on ERP changes during VSWM activation in HC and aMCI patients. On the basis of the above-mentioned theory, we hypothesized that: First, aMCI patients showed reduced P300 amplitude and delayed P300 latency compared to HC; Second, P300 amplitude and latency was correlated with information processing speed and executive function; Third, the P300 component with lower amplitude and delayed latency was observed in APOE ε4 carriers with respect to non-carriers in non-demented participants and aMCI patients; Forth, APOE ε4 carriers showed brain activation alteration compared to non-carriers.

## Materials and Methods

### Participants

In this study, a cohort of 39 aMCI patients and 43 HC were enrolled through community health screening, newspaper advertisements and a memory outpatient clinic (all participants were Chinese Han). The responsible Human Participants Ethics Committee of the Affiliated ZhongDa Hospital, Southeast University (2016ZDSYLL011.0) approved our study. All the participants provided written informed consents. Moreover, all the participants ranged from 55 to 80 years old and were right-handed.

### Neuropsychological Assessments

As described previously (Shu et al., 2016; Su et al., 2017), all participants underwent neuropsychological assessments, including Mini-Mental State Examination (MMSE), Mattis Dementia Rating Scale-2 (MDRS-2), Clinical Dementia Rating (CDR), Auditory Verbal Learning Test-Delayed Recall (AVLT-DR), the Rey–Osterrieth Complex Figure Test-Delayed Recall (ROCFT-DR), Trail Making Tests (TMT), and Stroop Test (ST), Digit Span Test, Symbol Digit Modalities Test, and the Clock Drawing Test to assess cognitive functions (general cognitive functions, episodic memory, executive function, information processing speed, and visual spatial domains).

### Inclusion Criteria and Exclusion Criteria

All aMCI patients met the diagnostic criteria (Petersen, 2004; Albert et al., 2011; Bai et al., 2011), which include: Subjective memory impairment confirmed by the participant and his informant; Objective memory performance documented by an AVLT-delayed recall score less than or equal to 1.5 standard deviation (SD) of age- and education-adjusted norms (cut-off of ≤ 4 correct responses on 12 items for patients with ≥8 years of education); MMSE score equal or greater than 24; CDR of 0.5; No or minimal impairment in activities of daily living; Absence of dementia or insufficient dementia to meet the NINCDS-ADRDA (National Institute of Neurological and Communicative Disorders and Stroke and the AD and Related Disorders Association) Alzheimer’s Criteria. Additionally, the controls had a CDR of 0, an MMSE score equal or greater than 26, and an AVLT-delayed recall score greater than 4 for participants with 8 or more years of education. Participants who had a history of neurological or psychiatric disorders, major medical illness, or severe visual or hearing loss were excluded from the study.

### APOE Genotyping

The process of APOE genotyping was on the basis of previous protocols (Shu et al., 2014, 2016). Each participant’s genomic deoxyribonucleic acid (DNA) was extracted, with a DNA direct kit, from 250-μL ethylenediaminetetraacetic acid (EDTA)-anticoagulated blood (Tiangen, China). A polymerase chain reaction-based restriction fragment length polymorphism (PCR-RFLP) assay detected alleles of rs7412 and rs429358, respectively. Eventually, the APOE genotype was determined by the haplotype of rs7412 and rs429358. Thus, the participants were further divided into four groups according to their APOE genotypes, as displayed in **Table 1**. Each group was identified as HC-APOE ε4-, HC-APOE ε4+, aMCI-APOE ε4-, and aMCI-APOE ε4+.

**Table 1:** Demographic data and neuropsychological performance for all participants.

	HC		aMCI		F or χ^2^	*p*
	APOE ε4- (*n* = 25)	APOE ε4+ (*n* = 18)	APOE ε4- (*n* = 27)	APOE ε4+ (*n* = 12)		
Age (years)	70.16 (5.65)	70.17 (5.26)	71.33 (6.32)	71.17 (5.39)	0.259	0.855^$^
Gender (Male/Female)	12/13	8/10	17/10	8/4	2.213	0.474^%^
Education (years)	12.28 (2.57)	12.28 (3.52)	10.87 (2.98)	10.67 (2.94)	1.667	0.181^$^
MMSE scores	28.68 (1.11)	28.06 (1.55)	27.15 (1.88)^a^	26.92 (2.64)	4.503	0.006^$∗^
MDRS-2 scores	137.28 (2.87)	137.94 (3.67)	134.70 (5.70)	135.17 (4.71)	2.694	0.052^$^
CDR	0	0	0.5	0.5	-	-
**Composite *Z* scores of each cognitive domain**						
Episodic memory	0.54 (0.49)	0.45 (0.61)	–0.48 (0.63)^a^	–0.67 (0.56)^b^	22.537	<0.001^$∗^
Information processing speed	0.16 (0.85)	0.36 (0.73)	–0.23 (0.56)	–0.18 (0.81)	2.941	0.038^$∗^
Executive function	0.22 (0.61)	0.31 (0.71)	–0.25 (0.48)^a^	–0.22 (0.58)	4.057	0.010^$∗^
Visuospatial function	0.16 (0.61)	0.04 (0.57)	–0.16 (0.76)	–0.21 (0.42)	1.602	0.196^$^

*Significant difference was indicated between four groups.*

*Data are presented as mean ± SD.*

*^$^*p*-values were obtained by one-way ANOVA analysis. ^%^*p*-value was obtained by Fisher’s exact probability tests. ^∗^Significant differences were found in MMSE scores, episodic memory, information processing speed and executive function among the four groups. ^a^*Post hoc* Bonferroni’s multiple comparison test further revealed the source of ANOVA difference (*p* < 0.05, HC-APOEε4- vs. aMCI-APOEε4-). ^b^*Post hoc* Bonferroni’s multiple comparison test further revealed the source of ANOVA difference (*p* < 0.05, HC-APOE ε4+ vs. aMCI-APOE ε4+). aMCI, amnestic mild cognitive impairment; ANOVA, analysis of variance; APOE, apolipoprotein E; CDR, Clinical Dementia Rating; HC, healthy controls; MDRS-2, Mattis Dementia Rating Scale-2; MMSE, Mini-Mental State Examination; SD, standard deviation.*

### Procedure and Stimuli

**FIGURE 1:**
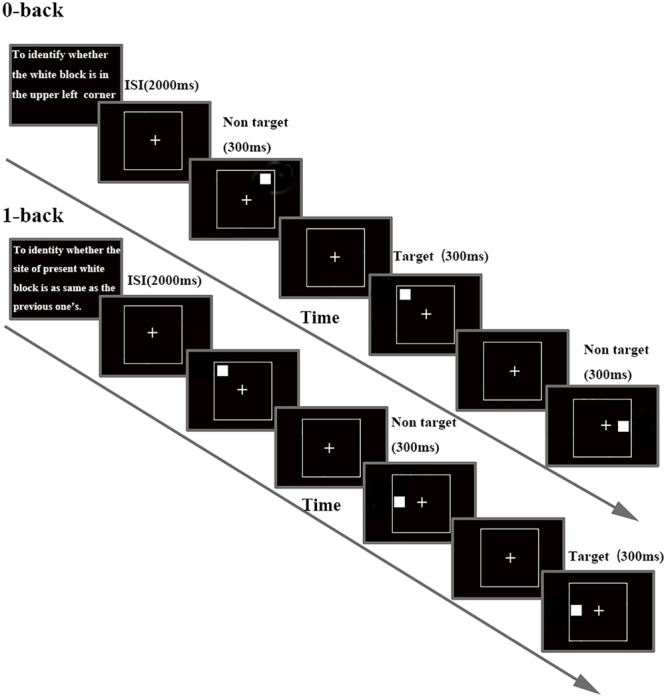
**Schematic diagram of the N-back task, separately for each task load.** Details about timing for stimulus and inter stimulus interval (ISI) are included.

Participants sat in front of a 17 inch computer screen (refresh rate 75 Hz) placed at a distance of 70 cm from their eyes in a quiet, dimly lit, sound-proofed room, with an ambient temperature of about 24°C (Chen et al., 2013; Jiu et al., 2013). Stimuli and task were controlled with E-Prime 2.0 software (Psychology Software Tools Inc., Pittsburgh, PA, United States). The investigation used a visual N-back task (0- and 1-back task, see **Figure 1**) to assess WM. Each block started with the statements of instructions. Participants were asked to minimize head, body movements and to keep their eyes fixated on a cross presented in the middle of the screen throughout the stimulus presentation. A continuous stream of white block (size: 2.6 × 2.6 degree at 70 cm from the face) was presented, one white block per frame, for 300 ms each. Inter stimulus interval (ISI) were 2000 ms in both studied conditions. Participants were asked to respond using their dominant hand. Each white block was randomly positioned in one of eight possible locations (both ends of horizontal *X*-axis, vertical *Y*-axis and the lower and upper position of both diagonals). In 0-back load, participants were asked to recognize whether a white block appeared on the upper left side of the screen. In 1-back load, participants had to identify whether the current stimulus displayed in the same locations as the previous presentation. Participants had to distinguish between targets and non-targets by pressing the button with their right index or middle finger. Response time (RT) and accuracy were recorded after pressing the button. Trials were presented in eight blocks (4 blocks for 0- and 1-back task, respectively); each block representing either the control (0-back) or WM conditions (1-back). Each block was composed of 20 trials with a 3:7 target/no target stimulus (Gevins and Cutillo, 1993). All the participants were instructed to respond as fast and as accurately as possible.

### EEG Acquisition and Analysis

Electroencephalogram (EEG) measurements were recorded with a BrainAmp MR portable ERP system (Brain Products GmbH; Munich, Germany) from 64 scalp electrodes (impedances < 5 kΩ; vertex reference; 500 Hz digitization). Electrodes were arranged according to the international extended 10–20 system. FCz electrode acted as an internal reference during online recording. All electrodes were re-referenced to an average reference for data off-line analysis. The electrooculogram (EOG) was positioned above and below the right eye (vertical EOG) and at the outer canthi of both eyes (horizontal EOG). Ocular artifact correction was performed with independent component analysis (Jung et al., 2000; Neuhaus et al., 2011). The EEG was band-pass filtered from 0.1 to 100 Hz (and a gain of 20,000). Data were analyzed with BrainVision Analyzer software 2.0 (Brain Products GmbH; Munich, Germany). In offline analyses, the EEG signal was band-pass filtered 0.01–30 Hz (Chen et al., 2014a,b). EEG signals with amplitude larger than ±100 μV were rejected (Chen et al., 2014c). To calculate the ERP, epochs of EEG were averaged offline from 100 ms pre stimulus to 700 ms post stimulus relative to a 100-ms pre stimulus baseline. The latency ranges were 300–500 ms for the P300. The peak amplitude and latency of the P300 component were acquired with peak detection process. On the basis of these studies (Sokhadze et al., 2008; Shishkin et al., 2011; Yu and Zhang, 2014), P300 variables were obtained at central–parietal and parietal electrodes.

### Statistical Analysis

The statistical analyses were implemented with SPSS 21.0 software. Statistical threshold was set at a *p* < 0.05.

#### Demographic and Neuropsychological Data

One-way analysis of variance (ANOVA) and Mann-Fisher’s exact probability tests were performed to compare the demographic data and neuropsychological performances between APOE ε4 carriers and non-carriers in aMCI patients and HC. Bonferroni’s *post hoc* test was used to detect the source of ANOVA difference.

#### Behavioral Analysis

Accuracy and RT were analyzed with a repeated measures analysis of covariance (RMANCOVA) with appropriate Greenhouse–Geisser corrections. Each RMANCOVA was conducted using memory loads (0-back and 1-back), stimulus types (target stimulus and non-target stimulus) as within-subject factors, and groups (aMCI patients and HC), APOE genotypes (APOE ε4 carriers and APOE ε4 non-carriers) as between-subject factors. Age, gender, years of education were included as covariates. *Post hoc* comparisons were adjusted using the Bonferroni correction.

#### ERP Analysis

Mean peak amplitude and latency were subjected to RMANCOVA with memory loads and electrode locations (CP1, CPz, CP2, P1, Pz, and P2) as within-subject factors, groups, APOE genotypes as between-subjects factors. Age, gender, years of education were included as covariates. *Post hoc* comparisons were adjusted using the Bonferroni correction.

Next, the mean P300 amplitudes and latencies for each task load were obtained by averaging across electrodes. Pearson Correlation was conducted between P300 amplitude/latency and neuropsychological performance (general cognitive functions, episodic memory, executive function, information processing speed, and visual spatial domains) partialing out the effect of age, gender, education, and APOE genotypes in HC and aMCI patients, respectively.

#### Source Localization Analysis

The standardized low-resolution brain electromagnetic tomography analysis (sLORETA) is an efficient functional imaging method for source localization and widely used in ERP studies (Pandey et al., 2012). It was implemented widely in WM studies (Suchan et al., 2005; Keeser et al., 2011). The sLORETA version in our investigation received considerable validation in previous studies (Pascual-Marqui, 2002; Pandey et al., 2012; Li et al., 2016), available at: http://www.uzh.ch/keyinst/loreta.htm. The cortex was modeled as a collection of volume elements (voxels) in the digitized Montreal Neurological Institute (MNI) coordinates corrected to the Talairach coordinates (Pascual-Marqui, 2002). The electrode coordinates were created from the 61 electrode locations. A transformation matrix was created with the electrode coordinates. In the study, the averaged waveforms (for all 400 time samples, from 100 ms pre stimulus to 700 ms post stimulus) for each participant were converted and saved into ASCII values. The contrast for the 1-back condition relative to the 0-back condition was selected for statistical study in order to derive statistical power by comparing the active task with the control task. To test for hypothesized differences in brain activation as a function of genotype, the contrast for the 1-back relative to 0-back was entered into an independent *t*-test analysis in sLORETA, with the APOE genotypes as the between-subject factors. Source localization was composed of two key parts: ERP analysis and LORETA analysis. During ERP analysis, we detected 1-back relative to 0-back difference between APOE ε4 carriers and non-carriers with *t*-statistic analysis. After view of the combined ERP (which is not an actual ERP), latency where maximum *t*-value occurred were attained for LORETA analysis. The sLORETA values for each participant were computed with ASCII values, electrode coordinates and transformation matrix, separately. Regarding the sLORETA analysis, 1-back relative to 0-back differences between two groups were compared in the time range of P300 component which showed difference in amplitude or latency. Finally, images were generated to localize these differences in the three dimensional (3-D) space within the brain.

## Results

### Demographic and Neuropsychological Results

Demographic and neuropsychological characteristics of participants are shown in **Table 1**. One way ANOVA indicated no significant differences between four groups in age, gender, and years of education. It detected significant differences between four groups in MMSE scores [*F*(3,78) = 4.503, *p* = 0.006], episodic memory [*F*(3,78) = 22.537, *p* < 0.001], information processing speed [*F*(3,78) = 2.941, *p* = 0.038] and executive function [*F*(3,78) = 4.057, *p* = 0.010], whereas no significant differences were indicated in Mattis Dementia Rating Scale-2 (MDRS-2) scores and visuospatial function between four groups. *Post hoc* analysis indicated significantly decreased MMSE scores, episodic memory and executive function in aMCI-APOE ε4- participants compared to HC-APOE ε4- participants. In addition, HC-APOE ε4+ participants showed better episodic memory compared to aMCI-APOE ε4+ participants.

### Behavioral Results

**Table 2** showed accuracy and correct RTs in each memory load condition (0- and 1-back) for target and non-target stimuli for aMCI patients and HC with different APOE genotypes.

**Table 2:** Behavioral data [accuracy and response time (RT)] for healthy controls and aMCI patients with different APOE ε4 status.

Condition	Stimulus	HC		aMCI	
		APOE ε4- (*n* = 25)	APOE ε4+ (*n* = 18)	APOE ε4- (*n* = 27)	APOE ε4+ (*n* = 12)
**Accuracy**					
0-back	Non-target	0.95 (0.04)	0.91 (0.07)	0.90 (0.08)^a^	0.86 (0.13)
	Target	0.88 (0.11)	0.83 (0.08)	0.78 (0.18)	0.72 (0.14)^b^
1-back	Non-target	0.82 (0.10)	0.77 (0.09)	0.74 (0.14)^a^	0.70 (0.20)
	Target	0.83 (0.10)	0.78 (0.09)	0.74 (0.18)	0.60 (0.18)^b^
**Response time**					
0-back	Non-target	640.96 (117.98)	645.22 (58.44)	663.57 (119.89)	768.61 (206.29)^b^
	Target	682.02 (118.39)	699.71 (93.11)	713.53 (92.59)	787.60 (172.46)^b^
1-back	Non-target	643.33 (122.62)	665.64 (62.34)	787.74 (169.42)	838.15 (197.73)^b^
	Target	759.40 (158.11)	817.06 (107.08)	941.52 (187.56)	988.89 (180.22)^b^

*Data are presented as mean ± SD.*

*^a^*Post hoc* tests by Bonferroni’s analysis further revealed the source of ANCOVA difference (*p* < 0.05, HC-APOE ε4- vs. aMCI-APOE ε4-). ^b^*Post hoc* tests by Bonferroni’s analysis further revealed the source of ANCOVA difference (*p* < 0.05, HC-APOE ε4+ vs. aMCI-APOE ε4+). aMCI, amnestic mild cognitive impairment; ANCOVA, analysis of covariance; APOE, apolipoprotein E; HC, healthy controls; SD, standard deviation.*

RMANCOVA showed a significant main effect of groups on accuracy [*F*(1,78) = 10.443, *p* = 0.002], whereas it indicated no significant main effect of APOE genotypes on accuracy. *Post hoc* analysis detected that MCI-APOE ε4- participants showed decreased accuracy with non-target stimuli in both two conditions (0-back and 1-back) with respect to HC-APOE ε4- participants (all *p* < 0.05, see **Table 2**). Additionally, *post hoc* analysis showed reduced accuracy for aMCI-APOE ε4+ participants compared to HC-APOE ε4+ participants with target stimulus in both two conditions (0-back and 1-back) (all *p* < 0.05, see **Table 2**).

RMANCOVA showed a significant main effect of groups on RT [*F*(1,78) = 9.506, *p* = 0.003], whereas it revealed no significant main effect of APOE genotypes on RT. *Post hoc* analysis detected that aMCI-APOE ε4+ participants showed prolonged RT compared to HC-APOE ε4+ participants with non-target and target stimuli in both two conditions (0-back and 1-back) (all *p* < 0.05, see **Table 2**).

### ERP Results

**FIGURE 2:**
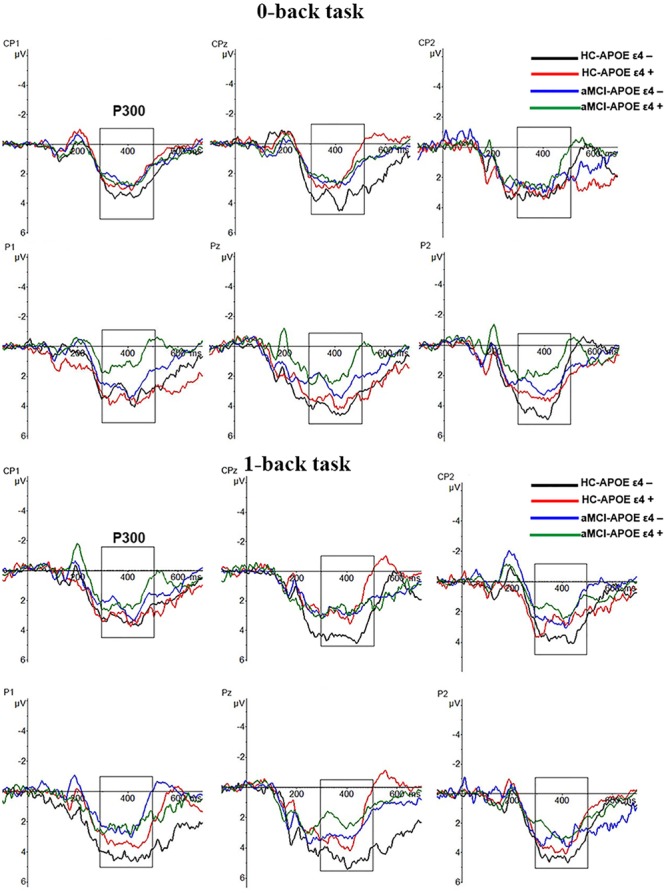
**Grand-average event-related potential (ERP) waveforms of the 0-back and 1-back task for HC-APOE ε4–, HC-APOE ε4+, aMCI-APOE ε4–, and aMCI-APOE ε4+ participants.** Grand-average ERP at the central–parietal (CP1, CPz, and CP2) and parietal (P1, Pz, and P2) electrodes for HC-APOE ε4– participants in 0- and 1-back task (black line), HC-APOE ε4+ participants in 0- and 1-back task (red line) and aMCI-APOE ε4– participants in 0- and 1-back task (blue line), aMCI-APOE ε4+ participants in 0- and 1-back task (green line). Dashed squares on each graph represent the time windows used for analyses of the P300 component. Abbreviations: aMCI, amnestic mild cognitive impairment; APOE, apolipoprotein E; HC, healthy controls.

P300 amplitude and latency were assessed at central–parietal (CP1, CPz, and CP2) and parietal (P1, Pz, and P2) electrodes, where the component was maximal (see **Figure 2**). RMANCOVA indicated main effects of groups and APOE genotypes on P300 amplitude [groups: *F*(1,78) = 9.160, *p* = 0.004; APOE genotypes: *F*(1,78) = 5.077, *p* = 0.028]. *Post hoc* analysis detected decreased P300 amplitude in HC-APOE ε4+ participants compared to HC-APOE ε4- participants at P2 electrode in 0-back task and CPz electrode in 1-back task (all *p* < 0.05, see **Table 3**). In addition, aMCI-APOE ε4+ participants showed decreased P300 amplitude compared to aMCI-APOE ε4- participants at P1 electrode in 0-back task and CP2 electrode in 1-back task (all *p* < 0.05, see **Table 3**). Moreover, *post hoc* analysis indicated that P300 amplitude was diminished for aMCI-APOE ε4- participants compared to HC-APOE ε4- participants at CPz, P2 electrodes in 0-back task and CPz, P1, P2 electrodes in 1-back task. Additionally, aMCI-APOE ε4+ participants showed reduced P300 amplitude with respect to HC-APOE ε4+ participants at P1 electrode in 0-back task and CP2, P2 electrodes in 1-back task. RMANCOVA indicated no significant main effects of groups or APOE genotypes on P300 latency.

**Table 3:** Event-related potential (ERP) data (P300 amplitude) for HC and aMCI patients with different APOE ε4 status.

Task	Site	HC		aMCI	
		APOE ε4- (*n* = 25)	APOE ε4+ (*n* = 18)	APOE ε4- (*n* = 27)	APOE ε4+ (*n* = 12)
0-back	CP1	3.69 (2.07)	3.23 (0.42)	3.16 (3.00)	2.44 (1.62)
	CPz	4.11 (1.63)	3.17 (0.68)	3.03 (1.82)^c^	2.45 (1.61)
	CP2	3.23 (1.69)	3.16 (0.87)	2.97 (1.64)	2.35 (1.66)
	P1	3.84 (2.37)	3.54 (1.01)	3.22 (1.80)	2.03 (1.78)^b,d^
	Pz	4.42 (2.25)	3.50 (0.91)	3.31 (1.77)	2.59 (2.56)
	P2	4.89 (2.02)	3.11 (1.00)^a^	3.04 (2.10)^c^	2.34 (1.96)
1-back	CP1	3.61 (2.14)	3.34 (0.65)	2.98 (3.38)	2.42 (1.59)
	CPz	4.63 (2.00)	3.21 (1.21)^a^	2.62 (1.80)^c^	2.53 (1.78)
	CP2	3.93 (1.92)	3.60 (1.12)	3.34 (2.07)	2.31 (1.56)^b,d^
	P1	4.49 (2.58)	3.24 (1.07)	3.00 (1.93)^c^	2.49 (2.10)
	Pz	5.11 (2.34)	3.43 (0.93)	3.23 (1.89)	2.54 (1.39)
	P2	4.52 (2.34)	3.71 (1.26)	3.53 (2.28)^c^	2.54 (1.74)^d^

*Data are presented as mean ± SD.*

*^a^*Post hoc* tests by Bonferroni’s analysis further revealed the source of ANCOVA difference (*p* < 0.05, HC-APOE ε4- vs. HC-APOE ε4+). ^b^*Post hoc* tests by Bonferroni’s analysis further revealed the source of ANCOVA difference (*p* < 0.05, aMCI-APOE ε4- vs. aMCI-APOE ε4+). ^c^*Post hoc* tests by Bonferroni’s analysis further revealed the source of ANCOVA difference (*p* < 0.05, aMCI-APOE ε4- vs. HC-APOE ε4-). ^d^*Post hoc* tests by Bonferroni’s analysis further revealed the source of ANCOVA difference (*p* < 0.05, aMCI-APOE ε4+ vs. HC-APOE ε4+). aMCI, amnestic mild cognitive impairment; ANCOVA, analysis of covariance; APOE, apolipoprotein E; ERP, event-related potential; HC, healthy controls; SD, standard deviation.*

**FIGURE 3:**
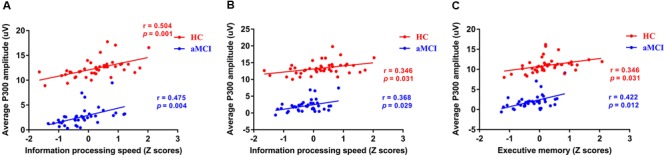
**Scatterplots illustrating the correlation between P300 amplitude and *Z* scores of information processing speed in 0-back task (A)**, *Z* scores of information processing speed in 1-back task **(B)**, and *Z* scores of executive function in 1-back task **(C)** in healthy controls and aMCI patients partialing out the effect of age, gender, years of education, and APOE ε4 status. Red circles represents HC. Blue circles represents aMCI patients. Abbreviations: aMCI, amnestic mild cognitive impairment; APOE, apolipoprotein E; HC, healthy controls.

Based on the results of ERP components analysis, the correlation between cognitive assessment score and ERP components parameters (P300 amplitude) partialing out the effect of age, gender, years of education, and APOE genotype was calculated in HC and aMCI patients, respectively. It revealed positive correlations between P300 amplitude and information processing speed in both 0-back and 1-back tasks (0-back task: *r* = 0.504, *p* = 0.001 for HC and *r* = 0.475, *p* = 0.004 for aMCI patients, respectively; 1-back task: *r* = 0.346, *p* = 0.031 for HC and *r* = 0.368, *p* = 0.029 for aMCI patients, respectively). Moreover, a significantly positive correlation was revealed between P300 amplitude in 0-back task and executive function (*r* = 0.346, *p* = 0.031 for HC and *r* = 0.422, *p* = 0.012 for aMCI patients) (see **Figure 3**).

### Source Localization Analysis

**FIGURE 4:**
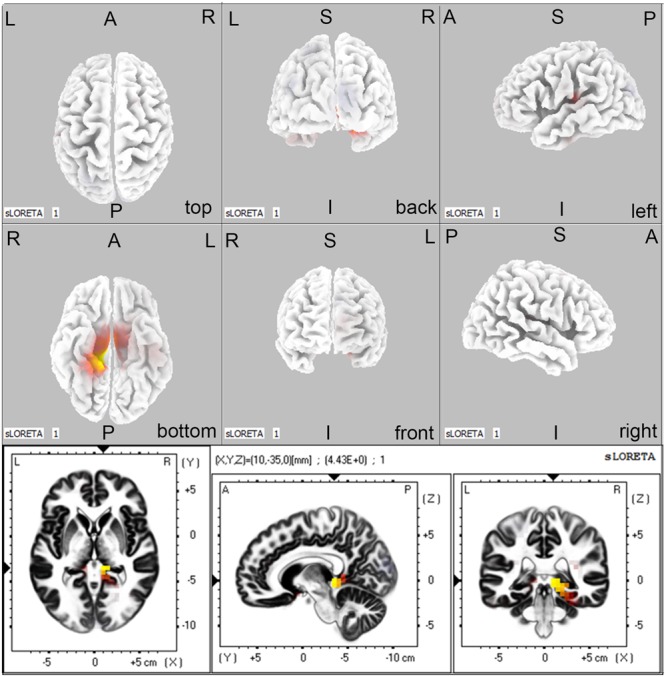
**The sLORETA images showing statistical differences between aMCI-APOE ε4+ and aMCI-APOE ε4– group (three dimensional-view and slice-view) during the P300 time range.** The three slice-view images below located the maximal difference between aMCI-APOE ε4+ and aMCI-APOE ε4– group (MNI coordinates *x, y, z* = 10, –35, 0). Activation maxima are color coded in yellow. These images represent greater brain activation in the right PHG in APOE ε4 carriers with respect to non-carriers in aMCI patients. Abbreviations: aMCI, amnestic mild cognitive impairment; APOE, apolipoprotein E; MNI, Montreal Neurological Institute; PHG, parahippocampal gyrus; sLORETA, standardized low-resolution brain electromagnetic tomography analysis.

Regarding the brain activation during VSWM, the differences between groups were displayed in **Table 4**. Brain regions that showed activation difference at <0.05 level were showed in **Figure 4** (activation maxima are color coded in yellow). It showed greater brain activation in the right parahippocampal gyrus [PHG, Brodmann area (BA) 27] in aMCI-APOE ε4+ compared to aMCI-APOE ε4- group during P300 time range (see **Table 4** and **Figure 4**). However, the difference in cortex activation between HC- APOE ε4+ and HC-APOE ε4- during P300 time range did not reach a significant level during P300 time range (see **Table 4**).

**Table 4:** Comparison of participants with different APOE ε4 status in sLORETA in HC and aMCI patients, respectively (voxels showing maximal difference).

Items	MNI coordinates			Brodmann area	Brain region	Log of ratio of average	*p*-value
	*x*	*y*	*z*				
HC- APOE ε4+ > HC-APOE ε4-	15	10	55	6	Right cerebrum, frontal lobe, superior frontal gyrus	–3.25	0.2664
aMCI-APOE ε4+ > aMCI-APOE ε4-	10	–35	0	27	Right cerebrum, limbic lobe, parahippocampal gyrus	4.40	0.0323

*aMCI, amnestic mild cognitive impairment; APOE, apolipoprotein E; HC, healthy controls; MNI, Montreal Neurological Institute; sLORETA, standardized low-resolution brain electromagnetic tomography analysis.*

## Discussion

The current study reported that aMCI patients showed reduced accuracy, delayed mean correct RT and decreased P300 amplitude at central–parietal and parietal electrodes compared to HC. P300 alteration is the most widely investigated and best understood component change in ERP studies on AD patients. P300 component represents different cognitive processes during different tasks (Kirschner et al., 2015). P3b is an important signature of WM processes (Linden, 2005). Consistent with our investigation, previous studies have shown P300 amplitude reduction during WM task in aMCI patients (Newsome et al., 2013; Li et al., 2016). These studies indicate that decreased P300 amplitude could be a sensitive biomarker for identifying aMCI patients. Moreover, our study detected that P300 amplitude was related to information processing speed and executive function in both HC and aMCI patients. Brown et al. (2012) suggested that information processing affects ERP results via speed of encoding. In addition, Miyake et al. (2001) indicated that central executive function plays a critical role in WM. In summary, P300 amplitude could predict VSWM deficits in aMCI patients.

Notably, P300 amplitude significantly differed between APOE ε4 carriers and non-carriers in cognitively intact adults, distinct from the lack of performance difference between different APOE ε4 status. Moreover, according to our study, P300 amplitude declined prior to task performance change, supporting the viewpoint that P300 amplitude alteration might be a sensitive index for detecting VSWM deficits in APOE ε4 carriers. However, sLORETA results showed no significant brain activation difference between different APOE ε4 status during P300 time range. A fMRI study with VSWM task also reported a similar result (Alichniewicz et al., 2012), but it was inconsistent with another fMRI investigation, that detected lesser neural activation in the MTL during VSWM task in APOE ε4 carriers compared to non-carriers in HC (Borghesani et al., 2008). In addition, another fMRI study reported that APOE ε4 carriers in cognitively intact adults showed greater brain activation in the medial frontal cortex and PHG during WM task compared to APOE ε4 non-carriers (Filbey et al., 2010). Moreover, a fMRI study indicated that APOE ε4 carriers showed greater brain activation in the bilateral medial frontal and parietal regions and the right dorsolateral prefrontal cortex with respect to APOE ε4 non-carriers during WM (Wishart et al., 2006). Heterogeneity of included individuals and differences in tasks might be the sources of inconsistence. It was reported that APOE ε4 allele had a dose effect on AD risk (Corder et al., 1993; Hostage et al., 2013). In addition, a recent meta-analysis indicated that APOE 3/4 allele status had no effect on global cognitive ability, including visuospatial skills in cognitively healthy individuals (Wisdom et al., 2011). However, due to the limitation of sample size, the dose effect could not be explored in the investigation. Interestingly, APOE ε4 allele showed no effect on brain activation in cognitively intact adults during visuospatial N-back WM tasks (the present study and Alichniewicz et al.’s study), whereas brain activation alteration presented between APOE genotypes during other VSWM tasks [e.g., delayed match to sample (DMS) tasks] (Borghesani et al., 2008). Moreover, the different biological backgrounds of fMRI and sLORETA might provide additional explanation for the source localization difference (Herrmann and Debener, 2008). Blood oxygenation level-dependent (BOLD) signal reflects blood oxygenation changes associated with neural activation (mainly presynaptic) and is based on metabolic processes (Logothetis and Pfeuffer, 2004), whereas ERP corresponds to electrical neuronal activation (mainly postsynaptic). Additionally, a disadvantage of ERP is the limitation of deep brain monitoring (Rusiniak et al., 2013). As proposed above, the different biological characters and brain monitoring depth might explain the source localization difference acquired from LORETA and fMRI. In summary, P300 amplitude might sensitively assess the WM deficits for AD high-risk participants.

Moreover, aMCI-APOE ε4 carriers showed reduced P300 amplitude compared to non-carriers, whereas the two groups performed tasks at the same level. Laczó et al. (2009) demonstrated more profound SWM deficits in aMCI-APOE ε4 carriers compared to non-carriers. Additionally, our sLORETA investigation revealed significantly enhanced brain activation in the right PHG in APOE ε4 carriers compared to non-carriers during P300 time range. It was inconsistent with a fMRI study during a VSWM task, that indicated no significant brain activation difference between different APOE ε4 status in aMCI patients (Alichniewicz et al., 2012). Both Alichniewicz et al.’s (2012) study and the present study applied visuospatial N-back WM. Heterogeneity of included individuals and different biological backgrounds of fMRI and sLORETA might provide an interpretation for the inconsistent results. Right PHG is a part of MTL, which is composed of hippocampus, entorhinal, perirhinal, and parahippocampal cortex. MTL is a critical region for long-term memory. Recent findings revealed that MTL is essential for relational processing to form WM, related to encoding of WM and could predict subsequent recall (Olson et al., 2006a,b; Hartley et al., 2007; Axmacher et al., 2008; Garrett et al., 2011). In the present study, APOE ε4 carriers showed significantly enhanced brain activation in the right PHG compared to non-carriers, whereas no significant task performance difference was indicated between APOE ε4 carriers and non-carriers. The situation might result from neural compensation. Neural compensation refers to recruitment of additional, compensatory networks to accomplish the task after primary task-related networks afflicted by pathology (Barulli and Stern, 2013). Several fMRI studies are also consistent with the neural compensation hypothesis in older adults at risk for AD, although none specifically investigated the VSWM (Bookheimer et al., 2000; Bondi et al., 2005; Bassett et al., 2006; Han et al., 2007). Investigations on patterns of association between the right PHG activation and VSWM task performance in different APOE genotypes might further demonstrate the neural compensation hypothesis. However, due to the limitation of LORETA on source localization, the present studies could not provide more evidence for the neural compensation hypothesis. According to our study, reduced P300 amplitude could act as a biomarker for VSWM deficits in aMCI-APOE ε4 carriers, and neural compensation mechanism might contribute to the maintenance of task performance.

There are a few limitations in the investigation. The present study did not explore the dose effect and the effect of specific genotype among APOE ε4 non-carriers due to the limitation of sample size. It was reported that different effect on cognition was indicated between APOE ε2 carriers and participants with APOE ε3/ε3 genotype (Benjamin et al., 1994). Source localization with LORETA lacks high spatial resolution, simultaneous ERP-fMRI could solve the problem. This study explored brain activation difference during low-load WM task exclusively; a high-load task could also be investigated to detect the impact of APOE ε4 status on WM with different task loads.

## Conclusion

The present investigation reported that aMCI patients showed reduced P300 amplitude compared to HC. Moreover, APOE ε4 status influenced P300 amplitude during VSWM in both non-demented adults and aMCI patients. However, the study did not detect task performance difference between APOE ε4 carriers and non-carriers, which might derive from neural compensation mechanism. Hence, the present investigation demonstrated that P300 amplitude could predict VSWM deficits in aMCI patients and contribute to early detection of VSWM deficits in APOE ε4 carriers compared to non-carriers.

## Author Contributions

L-HG, L-JG, and JC were in charge of EEG recording, ERP data acquisition and analysis. HS, ZW, DL, and Y-NY were in charge of patient enrollment, neuropsychological assessments. L-HGu had the major responsibility for preparing the paper and manuscript writing. Z-JZ, S-JL, and JC contributed to the design and plan of the present study. Z-JZ supervised the project.

## Conflict of Interest Statement

The authors declare that the research was conducted in the absence of any commercial or financial relationships that could be construed as a potential conflict of interest.
